# Potential Performance of Two New RT-PCR and RT-qPCR Methods for Multiplex Detection of Dengue Virus Serotypes 1–4 and Chikungunya Virus in Mosquitoes

**DOI:** 10.3390/cimb46100656

**Published:** 2024-09-30

**Authors:** Michel Kiréopori Gomgnimbou, Louis Robert Wendyam Belem, Etienne Bilgo, Miriam Félicité Amara, Zouera Laouali, Ali Ouari, Toussaint Bayala, Kobo Gnada, Raymond Kharlis Yao, Moussa Namountougou, Ibrahim Sangaré

**Affiliations:** 1 Centre d’Excellence Africain en Innovations Biotechnologiques pour l’Elimination des Maladies à Transmission Vectorielle (CEA/ITECH-MTV), Université Nazi BONI, Bobo-Dioulasso 01 BP 1091, Burkina Faso; louisro2007@yahoo.fr (L.R.W.B.); amaramiriamfelicite@gmail.com (M.F.A.); laoualiidi76@gmail.com (Z.L.); gnadakobodaniel@yahoo.fr (K.G.); karlhisyao@gmail.com (R.K.Y.); namountougou_d@yahoo.fr (M.N.); babaibrasangare@yahoo.fr (I.S.); 2 Laboratoire de Recherche, Centre MURAZ, Institut National de Santé Publique, Bobo-Dioulasso BP 10278, Burkina Faso; ouari_ali@yahoo.fr (A.O.); bayaladimitritoussaint@gmail.com (T.B.); 3 Institut Supérieur des Sciences de la Santé (IN.S.SA), Université Nazi BONI, Bobo-Dioulasso 01 BP 1091, Burkina Faso; 4 Institut de Recherche en Sciences de la Santé, Bobo-Dioulasso BP 545, Burkina Faso; bilgo02@yahoo.fr; 5 Unité de Formation et de Recherche en Sciences de la Vie et de la Terre (UFR SVT), Université Nazi BONI, Bobo-Dioulasso 01 BP 1091, Burkina Faso; 6 Centre Hospitalier Universitaire Sourô SANOU (CHUSS), Bobo-Dioulasso 01 BP 676, Burkina Faso

**Keywords:** multiplex RT-PCR, multiplex RT-qPCR, molecular detection, dengue virus, chikungunya virus, mosquitoes, Burkina Faso

## Abstract

Mosquitoes of the genus *Aedes* are the most important arthropod disease vector. Dengue virus (DENV) and Chikungunya virus (CHIKV) are the main arboviruses distributed throughout the world. Based on entomo-virological surveillance, appropriate public health strategies can be adopted to contain cases and control outbreaks. This study aims to show the potential performance of two new molecular methods for detecting DENV serotypes and CHIKV in mosquitoes. Mosquitoes were collected in urban and sylvatic areas of Bobo-Dioulasso, Burkina Faso, between July and August 2023. DENV and CHIKV were screened using new multiplex RT-PCR and RT-qPCR methods. A total of 2150 mosquitoes were trapped, consisting of 976 Aedes (959 *Ae. aegypti*, 6 *Ae. furcifer*, and 11 *Ae. vittatus*) and 1174 *Culex* sp. These were grouped into 39 pools, with each pool containing a maximum of 30 mosquitoes. Molecular screening revealed that 7.7% (3/39) of the pools were positive for DENV. Specifically, DENV-1 was detected in one pool (1/3), and DENV-3 was found in two pools (2/3). All pools tested negative for CHIKV. The overall minimum infection rate (MIR) of DENV in this study was 3.07 (95% CI: 2.24–19.86). This study shows the usefulness of our new molecular tools for the surveillance of DENV serotypes and CHIKV.

## 1. Introduction

Dengue and Chikungunya are vector-borne diseases caused by arboviruses belonging to the *Flaviviridae* and *Togaviridae* families, respectively. They are the most prevalent arboviruses identified globally [1,2]. Mosquitoes are the most important arthropod disease vector in the world [3]. There are an estimated 300 species of mosquitoes capable of disease transmission; however, *Aedes* (*Ae.*) and *Culex* mosquitoes are considered the most medically important mosquito vectors [4]. *Aedes aegypti* and *Aedes albopictus* have been reported to play major roles in the transmission of Dengue virus (DENV) and Chikungunya virus (CHIKV) to humans and animals [5]. In Africa, other species, including *Ae. furcifer*, *Ae. vittatus*, *Ae. fulgens*, *Ae. luteocephalus*, *Ae. dalzieli*, *Ae. vigilax*, *Ae. camptorhynchites*, *Culex annulorostris,* and *Mansonia uniformis,* have also been implicated [6]. Many factors influence the transmission of arboviruses; for example, climate change and globalization directly impact the spread of mosquitoes [7]. The tropical climate of Burkina Faso is suited to mosquito breeding. Also, people in Burkina Faso largely depend on water storage devices, which create most of the *Ae. Aegypti*-preferred larval water containers. In Burkina Faso, DENV is the main arbovirus responsible for outbreaks, resulting in the highest morbidities and mortalities [8,9,10]. CHIKV seroprevalence has also been reported [11], and in December 2023, an outbreak of CHIKV occurred in Burkina Faso, mainly in the region of Pouytenga, with 311 cases confirmed by PCR [12]. When DENV serotypes co-circulate with CHIKV, this can lead to an increase in the risk for more severe dengue forms [7]. To date, no specific antiviral drug and no effective vaccine are available against DENV and CHIKV [13]. Until an effective vaccine becomes available, vector control will continue to be the primary approach for managing the transmission of these arboviruses. The best approach to predicting when and where imminent DENV or CHIKV epidemics will occur is to conduct routine molecular monitoring using entomo-virological surveillance of arbovirus circulation in mosquitoes and humans. This allows for early detection of any serotype or genotype shift in a geographic area. Appropriate public health strategies can then be implemented to contain the cases and thereby control the infection [14,15,16]. Mosquito-based arboviral surveillance is important for understanding the dynamics of arbovirus disease transmission in a region [3].

Due to the low arbovirus infection rates in mosquito populations, entomo-virological surveillance needs to maximize sample sizes during trapping in order to increase detection probability [17]. Generally, real-time RT-PCR using TaqMan technology is used to detect arbovirus in mosquitoes. However, this method is expensive if used for surveillance with a large number of samples, especially in the least-developed countries like Burkina Faso. Thus, we previously developed a new, low-cost, one-step multiplex Reverse Transcription Polymerase Chain Reaction (mRT-PCR) and a one-step, real-time, multiplex RT-PCR (mRT-qPCR) with SYBR Green I for the rapid and simultaneous detection and genotyping of DENV and CHIKV for arbovirus diagnosis and surveillance in humans [18]. The aim of this study was to demonstrate the potential performance of these two new molecular methods for detecting DENV serotypes and CHIKV circulating in mosquitoes by screening in field-collected mosquitoes (urban and sylvatic areas) from the Hauts-Bassins region, Burkina Faso.

## 2. Materials and Methods

### 2.1. Study Area

**Figure 1:**
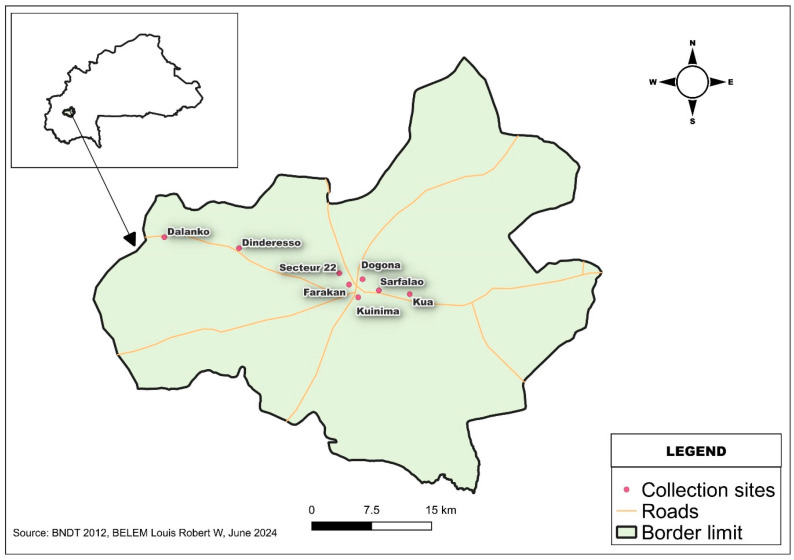
Map of the study area where mosquitoes were trapped.

This study was conducted in Bobo-Dioulasso from July to August 2023. Bobo-Dioulasso is the economic capital of Burkina Faso and is located in the region of Hauts-Bassins, in the western region of the country (11°11′00″ N, 4°17′00″ W), as indicated in Figure 1. The study area, like the rest of the western part of the country, has a South Sudanian climate characterized by two seasons: a rainy season from May to early October and a long dry season lasting around 6 months (October to April). The region of Hauts-Bassins vegetation is essentially dominated by wooded savannah and open forest and includes all subtypes from wooded savannah to grassy savannah. Mosquitoes were trapped in several sectors (Figure 1) of the urban area of Bobo-Dioulasso (Secteur 22, Farakan, Dogona, Kuinima, Sarfalao, and Kua) and the sylvatic areas (Dindéresso and Dalanko).

### 2.2. Larval and Adult Mosquito Collection

Mosquito larvae were collected from mosquito breeding habitats such as terracotta jars, plastic containers, metallic containers, and discarded tires. The collection was based on the predominance of *Aedes* larvae, distinguished from other larvae by their dark black color and curvilinear movements. Once the larvae had been identified, they were collected using a pipette and placed in plastic cups containing water. The presence of pupae was notified. Mosquito larvae were transferred to the insectary of Centre Muraz and reared to adult stages.

Adult mosquitoes were collected using mechanical aspirators. Collections were made in urban areas—mainly in households—and in forest areas—particularly near natural streams and rivers—to determine the sylvatic circulation of DENV and CHIKV vectors. The number of traps used was based on the abundance of mosquitoes at different collection sites according to the following criteria: (i) sites near or crossed by a river, which are favorable for mosquito multiplication; (ii) areas where living conditions are precarious, with intensive water storage in containers, which favors the multiplication of *Aedes* mosquitoes; (iii) areas near medical centers characterized by a large influx of people and the potential presence of *Aedes* mosquitoes.

Daytime collections were conducted to capture daytime-biting *Aedes* mosquitoes. Mosquitoes were put in cages and transported to the entomology laboratory of Centre Muraz for identification. Collected adult mosquitoes and adult mosquitoes from larvae were anesthetized by incubation at −20 °C for 5 min and identified using a microscope (Leica, S6E, Denaher, Heerbrudd, Switzerland) based on morphological characteristics. Only the genus *Aedes* was included in this study and stored in 1.5 mL micro-centrifuge tubes containing 200 µL of RNAlater (Thermo Fisher Scientific Baltics UAB, Vilnius, Lithuania) in pools of a maximum of 30 mosquitoes, according to genus, species, sex, and collection sites. Samples were stored at −20 °C prior to nucleic acid extraction and molecular screening of DENV and CHIKV at the Centre MURAZ molecular biology laboratory. We did not include *Culex* samples in the arbovirus screening as *Culex* are not involved in the transmission of Dengue and Chikungunya viruses.

### 2.3. RNA Extraction

All mosquito pools were first homogenized manually for nucleic acid extraction by using 200 µL of phosphate buffer saline (PBS) 1X (Sigma-Aldrich, Merck KGaA, Darmstadt, Germany. The homogenized mosquitoes were centrifuged for 5 min at 15,000× *g*, and 140 µL of supernatant was removed from each subsample for Ribonucleic acid (RNA) extraction using the QIAamp Viral RNA Mini Kit (Qiagen, Hilden, Germany) according to the manufacturer’s instructions [19]. Purified RNA was stored at −80 °C until further processing.

### 2.4. Molecular Screening of DENV1–4 and CHIKV

RNA extracted from mosquito samples was screened for DENV1–4 and CHIKV multiplex detection. Screening was carried out using the following master mix: the PrimeScript One Step RT-PCR Kit (Takara Bio-Inc, Shiga, Japan) for mRT-PCR and the one-step QuantiTect SYBR Green kit (Qiagen, Hilden, Germany) for mRT-q PCR. The primers used in this study and the protocol for these assays were previously described by Belem et al. [18]. mRT-PCR was performed using the SimpliAmp^TM^ thermal cycler (Applied Biosystems, Thermo Fisher Scientific, Waltham, MA, USA), and PCR products were then analyzed using gel electrophoresis. Real-time RT-PCR was performed using the CFX96 Real-Time System (BIO-RAD, Hercules, CA, USA).

All mosquito pools were screened for DENV1–4 and CHIKV using a commercial ZDC (Zika, Dengue, and Chikungunya) Multiplex one-step RT-PCR kit, which is considered the gold standard, and the results were compared with our new assays.

### 2.5. Data Management

The real-time RT-PCR data were analyzed using the CFX manager software version 3.1 provided by Bio-Rad; a sample was positive if the cycle threshold (C_t_) value was equal to or less than 33 cycles for DENV and CHIKV [18]. DENV serotyping, as well as the differentiation between DENV and CHIKV, were carried out using melting temperature (Tm) analysis. The arbovirus minimum infection rate (MIR) with a 95% confidence interval was determined using Excel 2016. The MIR uses the assumption that a positive pool contains only one infected mosquito. The map figure was created using QGIS version 3.26.3.

## 3. Results

### 3.1. Mosquito Collection and Identification

**Figure 2:**
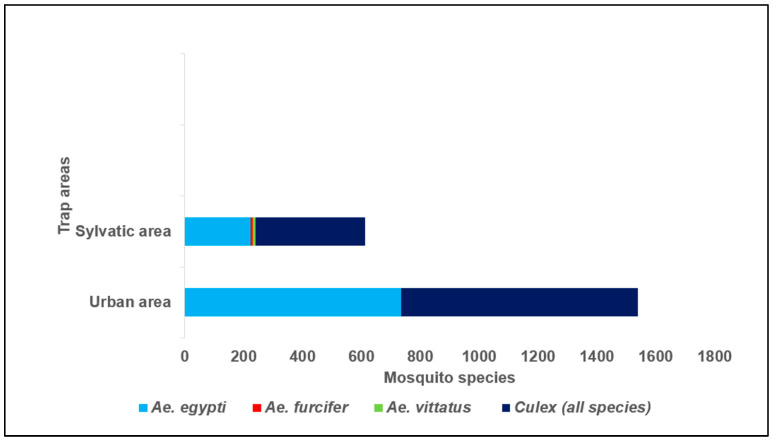
Distribution of mosquito species collected in urban and sylvatic areas of Bobo-Dioulasso from July to August 2023.

A total of 2150 mosquitoes were trapped in Bobo-Dioulasso from July to August 2023, including 976 (45.4%) *Aedes* spp. and 1174 (54.5%) *Culex* spp. The species and number of individuals collected were as expected in this study. Only the genus Aedes was included in the assays of this study. Of the 2150 mosquitoes collected, 69.0% (1484/2150) were collected in urban areas, while 31.0% (666/2150) were collected in sylvatic areas (Figure 2). Among the *Aedes* mosquitoes, 19.9% (195/976) were larvae that developed into adults after rearing. We identified 959 *Ae. aegypti* (476 males and 483 females), six *Ae. furcifer* (four males and two females), and 11 *Ae. vittatus* (seven males and four females). The repartition of the captured mosquitoes is displayed in Figure 2. All *Aedes* mosquitoes were divided into 39 pools with a maximum of 30 mosquitoes/pool, according to species, sex, and collection site.

### 3.2. Molecular Detection of Arboviruses in Mosquito Pools

**Figure 3:**
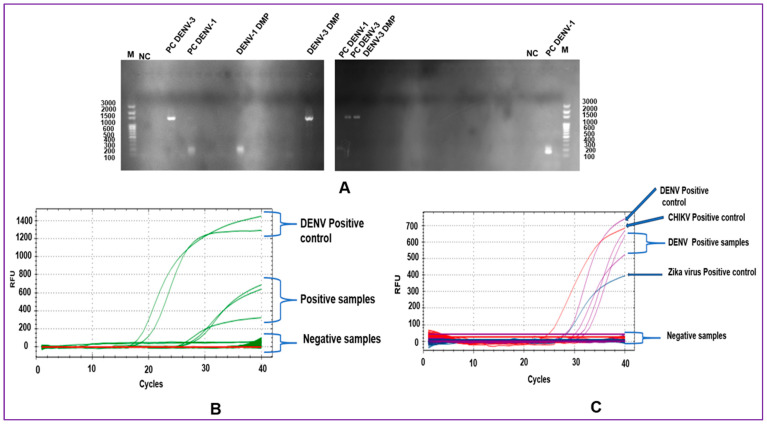
One-step multiplex amplification for DENV serotypes and CHIKV in mosquito samples. (**A**) Electrophoresis gel of multiplex RT-PCR amplification. M (100 bp DNA marker); DENV-1 (200 bp); DEN-3 (1359 bp); DMP: detect in mosquito pool; bp: base pair; NC: negative control. (**B**) Curves plots of multiplex RT-qPCR amplification. (**C**) Curves plots of ZDC (Zika, Dengue, and Chikungunya) from the Multiplex one-step RT-PCR kit.

All RNA of the 39 pools was screened using mRT-PCR and mRT-q PCR for DENV1–4 and CHIKV detection (Figure 3). From the 39 pools tested, 7.7% (3/39) (CI 95%: 2.1–26.52) were positive for DENV. All pools that were positive according to mRT-PCR were positive according to mRT-qPCR, and negative pools identified using the mRT-PCR method were negative using the mRT-qPCR method. Agreement between the mRT-PCR and mRT-qPCR methods was 100%. All pools shown to be positive by our assays (3/39) were also positive using the gold standard method (3/39) (CI 95%: 2.1–26.52). Negative pools (36/39) identified using our assays were also negative according to the gold standard method (36/39). Thus, the agreement between the gold standard method and the two new mRT-PCR and mRT-qPCR methods was 100%. Positive and negative predictive values, sensitivities, and specificities were 100%. However, we need a large size of positive pools for good 95% confidence interval values. DENV was detected only in *Ae. egypti* female pools from adult mosquito traps, and all positives were found in urban areas of Bobo-Dioulasso (Table 1). DENV was not detected in mosquitoes in the larval stage. CHIKV was not detected in the mosquito pools. Of the three positive pools, DENV-1 was detected in one pool (1/3) and DENV-3 in two pools (2/3). DENV-2 and DENV-4 were not detected. The MIR of DENV in urban areas was 4.08 (CI 95%: 3.01–25.91). The overall MIR of DENV in this study was 3.07 (95% CI: 2.24–19.86) (Table 1).

## 4. Discussion

In recent years, the distribution of vector-borne viruses has increased exponentially with climatic changes, migration, and globalization [7]. The distribution has taken place mainly in countries with tropical climates like Burkina Faso [7]. Entomo-virological surveillance plays an important role in arbovirus surveillance thanks to its efficacy in detecting early arbovirus circulation and implementing appropriate public health measures to contain the cases and thus control the infection [16]. This study was performed to screen DENV1–4 and CHIKV circulation in mosquitoes using new molecular methods previously developed by Belem et al., 2024 [18] and to show their potential and usefulness for arbovirus surveillance. The study took place in the context of the Dengue epidemic in Bobo-Dioulasso, Burkina Faso (data from Burkina Faso Ministry of Health weekly reports on potential epidemic diseases). The main mosquitoes included in this study were of the genus *Aedes*; among them, we identified three species. In urban areas, we identified only *Ae. egypti;* this explains why it is the main Dengue vector in Burkina Faso urban areas [20]. In Burkina Faso, many people store water for extended periods in various containers for routine household use. This practice provides permanent breeding sites for and a continuous source of *Ae. egypti* [21]. Other species, like *Ae*. *furcifer* and *Ae. vittatus,* were also identified in forest areas. Mosquito traps in these areas were deployed to detect DENV and CHIKV circulation in a sylvatic cycle. In this study, *Ae. Albopictus*, the second main vector of DENV and CHIKV, was not identified. To date, no study has identified these species in Burkina Faso [22].

In this study, our new molecular methods detected DENV in three pools of female *Ae. aegypti* from traps in urban areas, with an MIR of 4.08 (CI95%: 3.01–25.91). Serotyping has shown co-circulation of DENV-1 and DENV-3 in mosquitoes in Bobo-Dioulasso. These results demonstrate the efficacy of our new methods in detecting arbovirus in mosquitoes. Virological surveillance strategies for *Aedes* mosquitoes constitute a useful means for identifying high-risk areas for arbovirus transmission and an epidemic alert system [16]. Our new molecular methods will be useful for entomo-virological surveillance because they are sensitive, low-cost, and easy to use. The detection of DENV in mosquitoes followed a Dengue epidemic in Bobo-Dioulasso, as declared by the Director of Medical and Technical Services at CHUSS/Bobo-Dioulasso on 11 August 2016 and reported in the weekly reports of epidemiologic situations [12]. This study did not detect arbovirus in mosquitoes from collected larvae, but it is important to screen arboviruses in mosquitoes from larvae to determine vertical transmission. Vertical transmission can maintain the circulation of arbovirus in vector populations [23]. This situation could be the origin of a re-emergence of Dengue disease during the rainy season.

Mosquito traps in sylvatic areas were set up to determine the sylvatic circulation of DENV and CHIKV. Major arboviruses (DENV, CHIKV, Zika virus, and yellow fever virus) responsible for human infections originate in sylvatic cycle transmission, including vertebrate animals and wild mosquitoes [24]. Also, major arboviruses may transfer from the urban cycle to the sylvatic cycle and could hinder arbovirus eradication. Moreover, arbovirus could re-emerge anytime from a sylvatic cycle, creating outbreaks [25]. In this study, DENV serotypes and CHIKV were not detected in the sylvatic group. This can be explained by the fact that there was no active DENV or CHIKV sylvatic cycle during the collection period in sylvatic areas. Arbovirus emergence or re-emergence poses an imminent threat to public health; therefore, careful surveillance of arboviruses in both the human population and the sylvatic environment is crucial for implementing suitable public health measures to prevent outbreaks [16]. Further studies involving large-scale investigations of DENV serotypes and CHIKV circulation among wild mosquitoes, bats, and monkeys in various forest areas of Burkina Faso are needed to understand the arbovirus sylvatic cycle and to control it. The two new RT-PCR and RT-qPCR methods developed in the current study for the multiplex detection of arboviruses will certainly be straightforward and cost-effective methods t future studies.

## 5. Conclusions

This study demonstrates the performance and sensitivity of mRT-PCR and mRT-qPCR in detecting arboviruses in mosquito pools. It also confirms that *Ae. aegypti* is the primary species involved in DENV transmission in Bobo-Dioulasso, western Burkina Faso. These two new PCR methods are low-cost, easy to use, and can be employed for entomo-virological surveillance strategies. They provide important information for assessing the risk of arbovirus transmission and implementing disease control measures.

## Figures and Tables

**Table 1:** Results of mosquito pools tested for the distribution of Dengue and Chikungunya viruses within *Aedes* mosquitoes collected in urban and sylvatic areas of Bobo-Dioulasso from July to August 2023.

Area	Stage (Number of Mosquitoes)	DENV Positive Pools/All Pools	CHIKV Positive/Pools	DENV Serotyping				MIR (95% CI)
				DENV-1	DENV-2	DENV-3	DENV-4	
Urban area	Adult male (239)	0/29	0/29	0/3	0/3	0/3	0/3	0
	Adult female (301)	3/29	0/29	1/3	0/3	2/3	0/3	4.08 (3.01–25.91)
	Larvae (103 male + 92 female)	0/29	0/29	0/3	0/3	0/3	0/3	0
Sylvatic area	Adult male (150)	0/10	0/10	0/3	0/3	0/3	0/3	0
	Adult female (91)	0/10	0/10	0/3	0/3	0/3	0/3	0
Total		3/39	0/39	1/3	0/3	2/3	0/3	3.07 (2.24–19.86)

DENV, Dengue virus; CHIKV, Chikungunya virus; MIR, Minimum Infection Rate; CI, Confidence Interval.

## Data Availability

Data are contained within the article and Supplementary Materials (Supplementary Data).

## Supplementary Materials

The following supporting information can be downloaded at: https://www.mdpi.com/article/10.3390/cimb46100656/s1.
